# Simultaneous Quantitative Assessment of Ochratoxin A, Patulin, 5-Hydroxymethylfurfural, and Bisphenol A in Fruit Drinks Using HPLC with Diode Array-Fluorimetric Detection

**DOI:** 10.3390/foods9111633

**Published:** 2020-11-09

**Authors:** Norfarizah Hanim Hassan, Haneen Ibrahim Ahmad Al Othman, Nur Rabiatutadawiah Abdul Malek, Musfirah Zulkurnain, Bahruddin Saad, Yong Foo Wong

**Affiliations:** 1 School of Chemical Sciences, Universiti Sains Malaysia, Penang 11800, Malaysia; norfarizahhassan@gmail.com (N.H.H.); alothmanhanin@gmail.com (H.I.A.A.O.); tadakmph@gmail.com (N.R.A.M.); 2 Food Technology Division, School of Industrial Technology, Universiti Sains Malaysia, Penang 11800, Malaysia; musfirah.z@usm.my; 3 Fundamental and Applied Sciences Department, Universiti Teknologi PETRONAS, Seri Iskandar, Perak 32610, Malaysia; bahrudsaad@gmail.com

**Keywords:** high-performance liquid chromatography, core-shell column, mycotoxins, hydroxymethylfurfural, bisphenol A, fruit juice, fruit drinks

## Abstract

The analysis of regulated contaminants in fruit drinks often requires suitable validated and rapid analytical methods for cost-effective food control, and is of considerable interest among the fruit beverage industry. This study demonstrated a rapid and sensitive high-performance liquid chromatography approach for the simultaneous determination of ochratoxin A (OTA), patulin (PAT), 5-hydroxymethylfurfural (HMF), and bisphenol A (BPA) in various fruit drinks. The separations were achieved using a C18 core-shell column with both photo-diode array and fluorimetric detections connected in series. A gradient system consisting of methanol and 0.1% formic acid at a flow rate of 1.2 mL min^−1^, thermostated at 35 °C, provided fast elution with run time <9 min. Sample pretreatment was optimised to enable extraction of all analytes from fruit drink matrices. The optimised method was validated. Correlation coefficients of *R* > 0.99 were achieved with detection limits of 0.5 ng mL^−1^ (OTA), 1.1 ng mL^−1^ (PAT), 7.9 ng mL^−1^ (HMF), and 1.0 ng mL^−1^ (BPA). Recoveries ranged from 82% to 99%. Good relative standard deviations for intraday retention times (≤3.54%) and peak area (≤3.5%) were achieved. The developed multi-contaminants analysis method was successfully applied to determine OTA, PAT, HMF, and BPA in various fruit drinks.

## 1. Introduction

In recent years, the fast pace of modern lifestyles has led to changes in the consumers’ behavioural patterns in food preparation, consumption habits, and daily food intake to maintain a balanced and healthy diet. In particular, ready-to-drink pre-packaged fruit drinks have risen as a popular source of convenient and healthy beverage globally. Projections have indicated that the global fruit juices market will reach a volume of 50.6 billion litres by 2024 [1]. However, the growth of the markets has also raised food safety concerns among consumers on the possible exposure to contaminants, especially mycotoxins, endocrine-disrupting chemicals, and others [2]. The risk of such exposure is normally associated with possible production of contaminants during fruit storage, processing, and packaging [3,4].

**Figure 1:**
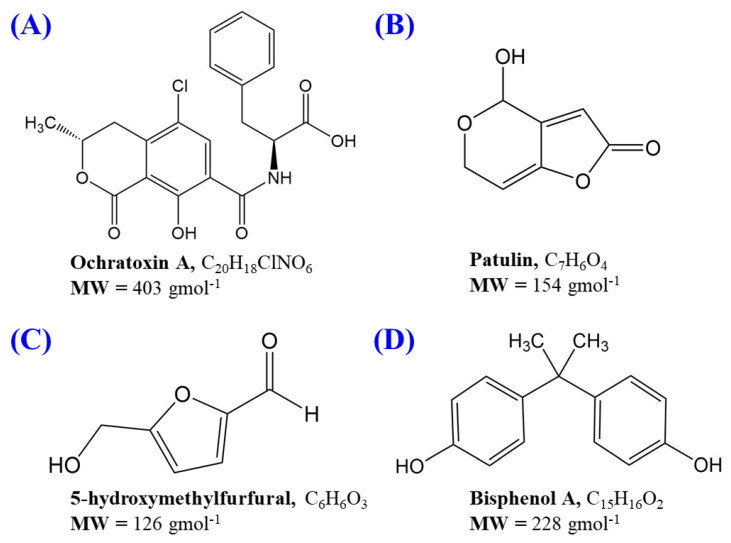
Molecular representation and molecular weight (MW) of (**A**) ochratoxin A (OTA); (**B**) patulin (PAT); (**C**) 5-hydroxymethylfurfural (HMF); (**D**) bisphenol A (BPA).

Mycotoxins are secondary metabolites naturally produced by certain types of microfungi or moulds. Acute consumption of these food-borne toxic compounds can cause severe illness and deaths in humans and animals. To date, several hundred mycotoxins have been identified, but the most commonly observed mycotoxins in fruits that present a concern to human health include ochratoxin A (OTA) and patulin (PAT). OTA (Figure 1A) is produced mainly by moulds from the genera *Aspergillus* and *Penicillium*, and commonly occur and distribute in several food products, including cereals, coffee beans, spices, dried fruits, grape juice, and others [5]. Tropical or subtropical regions with warm and humid climates are highly susceptible to OTA contaminations as a consequence of fungal growth [6]. Biologically, OTA displays hepatotoxic, teratogenic, carcinogenic, and immunosuppressive properties. Due to its chronic toxicity, the International Agency for Research on Cancer (IARC) has classified OTA as Group 2B (i.e., a possible human carcinogenic substance) [7]. As OTA is relatively stable, the Joint FAO/WHO Expert Committee on Food Additives (JECFA) has approved a provisional tolerable weekly intake for OTA of 100 ng kg^−1^ bw week^−1^, while the European Union Commission (EU) has established 2 µg kg^−1^ as the maximum permissible limits in grape juice, grape nectar, and its concentrates [8,9].

Besides OTA, patulin (PAT; Figure 1B), an unsaturated heterocyclic lactone, is one of the most studied mycotoxins in fruits and their processed products. PAT has been identified as a major causative agent of postharvest decay which is produced by a few species of fungi, including *Aspergillus*, *Byssochlamys*, *Gymnoascus*, *Paecilomyces*, and *Penicillium* [10,11]. These microfungi can be found in apples, apricots, peaches, oranges, tomatoes, and a range of their processed products. A few *Penicillium* spp. can grow even at below 5 °C, and the produced PAT is able to withstand thermal processing during the pasteurization process [11,12]. Clinically, PAT is known to exhibit genotoxic, immunotoxic, neurotoxic, teratogenic, and mutagenic properties, while acute PAT toxicity symptoms for humans include nausea, gastrointestinal disturbance, vomiting, and kidney failure [13,14,15]. Due to the evidence of its potential toxicity properties, the JECFA has suggested the provisional maximum tolerable daily intake for PAT of 0.2 µg kg^−1^ bw day^−1^ for young children and 0.4 µg kg^−1^ bw day^−1^ for adults [16]. The EU has regulated maximum permissible PAT levels in fruit juices, nectars, apple wine, and other fermented apple drinks at 50 µg kg^−1^, whilst the maximum level was set at 25 µg kg^−1^ in other solid apple products and 10 μg kg^−1^ for apple products for infants and young children [8].

In order to reduce the levels of mycotoxins and prolong the shelf life of the fruit drinks, thermal treatments are often applied during the manufacturing processes. However, such heating processes also promote the occurrence of non-enzymatic browning reactions (Maillard reactions and caramelisation), which mainly produce neo-formed contaminations, such as furan, 5-hydroxymethylfurfural (HMF; Figure 1C), and others. These by-products, particularly HMF (5-(hydroxymethyl) furan-2-carbaldehyde), have gained much interest due to their high toxicological potential to human. As fruit drinks contain a high level of carbohydrates, HMF can be developed during the heating process or prolonged storage [17,18]. The International Federation of Fruit Juice Processors (IFFJP) has suggested that the maximum level for HMF in fruit juice is 5–10 mg L^−1^ and 25 mg L^−1^ for fruit concentrates [19]. The EU and the Codex Alimentarius have established a maximum of 20 mg kg^−1^ HMF for juices made for children and 50 mg kg^−1^ in apple juice [20].

Apart from mycotoxins and furfurals, bisphenol A (BPA; Figure 1D) is another contaminant which is normally found in plastic-based packaging materials. BPA has been used industrially as the precursor to produce polycarbonate plastics which are widely used as packaging for food products. However, the polymer coating of the packaging can undergo polycarbonate hydrolysis, causing the leaching of BPA into the packaged food or fruits beverages. Studies have highlighted that the major BPA exposure pathway for human is through ingestion from contaminated food products [21]. Clinically, BPA has been identified as an endocrine disruptor which causes hormone imbalance in humans and could potentially disrupt major metabolic pathways [21,22,23]. Consumption of high doses of BPA may cause organ failure, leukaemia, and severe weight loss [22]. Previously, the European Food Safety Authority (EFSA) had recommended a tolerable daily intake of BPA of 50 µg kg^−1^ bw/day, however, due to the threat imposed by BPA exposure, it has been reduced to 4 µg kg^−1^ bw day^−1^ [24]. According to human BPA exposure data from a urinary BPA study conducted across seven Asian countries, the presence of BPA was found in ~94% of the analysed urine samples (concentration levels of <0.2–30.1 ng mL^−1^) [25].

Considering their potential occurrence in fruit drinks, various analytical approaches that include both sample preparation and chromatographic separation have been reported for the quantitation of OTA, PAT, HMF, and BPA either separately or in combinations. Due to the non-volatile nature of these compounds, liquid chromatography with different detection techniques has been the preferred method for their assessments. For gas chromatographic analyses, a derivatisation process is mandatory to improve the low volatility of these compounds. Although many methods have been developed for the determination of these food contaminants, studies on the simultaneous determination of OTA, PAT, HMF, and BPA have not been reported. In this paper, an improved HPLC-UV/FL method for the simultaneous extraction, separation, and quantification of these compounds in complex fruit beverages and drinks matrices are described. The method was validated, and its analytical practicality was demonstrated in the determination of these contaminants in different types of fruit drinks, including fruit juices and fruit concentrates commercially marketed in Malaysia.

## 2. Materials and Methods

### 2.1. Chemicals and Reagents

Patulin (PAT; ≥97%) was purchased from Romer Labs Diagnostic GmbH (Tulln, Austria). Bisphenol A (BPA; ≥99%; Toronto Research Chemicals Inc., North York, Ontario, Canada) was provided by Dr. Noorfatimah Binti Yahaya (Advanced Medical and Dental Institute, Penang, Malaysia). Ochratoxin A (OTA; ≥98%), 5-hydroxymethylfurfural (HMF; ≥99%), formic acid, Carrez I, potassium hexacyanoferrate (III) (~99%), and Carrez II, zinc acetate dihydrate (≥98%) were purchased from Sigma-Aldrich (St. Louis, MO, USA). HPLC grade acetonitrile (ACN) and ethyl acetate (EA) were purchased from Elite Advanced Materials Sdn. Bhd. (Selangor, Malaysia). Methanol (HPLC grade) was purchased from Fisher Scientific (Waltham, MA, USA). Chloroform was supplied by Merck (Darmstadt, Germany). Ultrapure water (resistivity: 18.2 MΩ cm^−1^) was produced using a Milli-Q system (Millipore, Burlington, MA, USA) and was used throughout the study. A nylon membrane filter (0.45 µm) was purchased from LabServ Sdn Bhd (Selangor, Malaysia). Sodium hydroxide pellets were obtained from Bendosen Laboratory Chemicals (Bendosen, Norway).

### 2.2. Fruit Beverage Samples

A total of 14 commercial fruit drinks and concentrates were randomly collected from local supermarkets in Penang, Malaysia. The samples collected included ambarella, apple, dates, grapes, guava, kiwi, lychee, mixed fruit, nutmeg, pineapple, plum (two samples), roselle, and soursop.

### 2.3. High-Performance Liquid Chromatographic System

Separation and quantification of analytes were carried out using the HPLC system (model 2695) from Waters Alliance (Waters Corporation; Milford, MA, USA). The instrument was equipped with a Waters (model 2998) photodiode array detector (PDA), a Waters (model 2475) multi λ fluorescence detector (FL), and an autosampler. The system was also equipped with an online degasser and a column oven. The PDA detector was configured at 284 nm and 276 nm to detect and quantify HMF and PAT, respectively. For fluorescence detection, BPA and OTA were identified and quantified using wavelengths of 275 nm (excitation) and 300 nm (emission), and 333 nm (excitation), and 443 nm (emission), respectively. The chromatographic separation was carried out using a solid core Kinetex^®^ EVO C18 column (150 cm × 4.6 mm i.d., 5 µm particle size; Phenomenex, CA, USA). The study was carried out in gradient-elution mode. The initial mobile phase was composed of methanol and acidified water (formic acid (0.1% *v/v*)) (18:82), retaining composition for 2 min, changed from 18% to 95% methanol at 8 min (retained 95% MeOH for 2 min) and lowered to 18% at 10.5 min (held for 2 min) with a flow rate of 1.2 mL min^−1^. The volume of the injected sample was 20 μL, and the column temperature was set at 35 °C.

### 2.4. Stock and Standard Solutions

Stock solutions of HMF (2000 µg mL^−1^), OTA (19.6 µg mL^−1^), and PAT (4 µg mL^−1^) were prepared in ultrapure water. BPA stock solution (1000 μg mL^−1^) was prepared with ultrapure water and acetonitrile with a ratio of 60:40, respectively. The individual stock standard solutions were sonicated and stored (4 °C). Standard solutions for the calibration curves were prepared by the appropriate dilution of the stock solutions with ultrapure water.

### 2.5. Preparation of Fruit Drink Samples

A miniaturised liquid–liquid extraction procedure with some modifications was employed [26,27]. The first clean-up method involved the addition of Carrez I (0.451 mol L^−1^) and Carrez II (1.339 mol L^−1^) solutions (500 μL) to the fruit drink sample (40 mL), followed by centrifugation. For fruit concentrates, appropriate dilution was made when the concentration of the analytes exceeded the linear ranges. The precipitate was then separated by decantation and the pH of resulting sample solutions was adjusted to pH ~8 using 5 M NaOH to eliminate excess Zn ions as Zn (OH)_2_. Prior to extraction, the purified sample was stored in a refrigerator (4 °C). All the samples (1 mL) were extracted twice using CHCl_3_ (1 mL) with vortexing (6 min), and the remaining water phase was subjected to additional extraction using 1 mL of ethyl acetate. The resulting organic phases were then evaporated to dryness under a gentle flow of nitrogen. The dried residue was immediately reconstituted in 400 μL of MeOH:water (30:70; *v/v*) mixture and syringe filtered with a 0.45-μm nylon membrane filter. The filtered solution was directly injected into the HPLC-UV/FL system.

### 2.6. Method Validation

Method validation parameters (linearity, limit of detection (LOD), limits of quantification (LOQ), repeatability, and recovery) were investigated. The linearity was investigated over the range of 0.5–50.0 ng mL^−1^ (OTA), 10–200 ng mL^−1^ (PAT), 10–1000 ng mL^−1^ (HMF), and 1–100 ng mL^−1^ (BPA). LOD values were estimated at an S/N ratio of 3.3, whereas LOQ was estimated at an S/N of 10. Intra-day precision was studied by analysing standard mixtures of OTA (2, 20, and 40 ng mL^−1^), PAT (20, 60, and 150 ng mL^−1^), HMF (40, 400, and 800 ng mL^−1^), and BPA (5, 40, and 80 ng mL^−1^) at three concentration levels, on the same day. The obtained results were expressed as relative standard deviation (% RSD). The recovery test was performed by spiking three concentration levels for OTA, PAT, HMF, and BPA at different spiked ranges; low concentration (2–20 ng mL^−1^), mid concentration (20–400 ng mL^−1^), and high concentration (45–800 ng mL^−1^) into a selected fruit drink sample.

### 2.7. Data Handling

Data acquisition and processing were performed using Empower 2 software (Waters Corporation, Milford, MA, USA). Acquired Empower 2 data were exported in CSV file format, followed by reconstruction using OriginPro 8 SR4 V8.0951 (B951) software (Origin, Northampton, MA, USA) to generate the chromatograms. Statistical analyses were performed using Microsoft Excel Version 2002 (Build 12527.21104; Microsoft Corporation, Redmond, WA, USA).

## 3. Results and Discussion

### 3.1. HPLC-DAD/FL Method Development

In the present work, an octadecyl silane (C18) core-shell column, which provides better chromatographic efficiency and generates flatter van Deemter curves compared to the C18 column packed with fully-porous particles, was chosen to effect the appropriate chromatographic separations [28]. The influences of chromatographic variables on solutes retention and separation were studied. Initially, the effect of different ratios of water (0–100%) and methanol (0–100%) or acetonitrile (0–100%) as mobile phases for the separation of OTA, PAT, HMF, and BPA were investigated. Results indicated that a composition of MeOH:H_2_O (18:82, *v/v*%) provided good resolution of the analytes. However, the analysis time was relatively long (>30 min) due to the late elution of BPA and OTA. To further shorten the analysis time, a gradient elution approach was initiated using 18% MeOH (held for 2 min; to promote separation of PAT and HMF), which progressively ramped to 95% MeOH (held for 6 min; to resolve OTA and BPA), then returned to 18% MeOH and equilibrated for 2 min. As OTA is a weak acid (pKa value~4.4), the mobile phases were added with formic acid (0.1% *v/v*) to prevent potential peak tailing and unspecific adsorption to the C18 column [29]. Good efficiency and shorter analysis time (by ~70%) were achieved by applying the gradient elution at a flow rate of 1.2 mL min^−1^.

Theoretically, elevated column temperature can reduce the viscosity of the mobile phase and increase the diffusion coefficient and mass transfer, which subsequently reduce peak broadening. Thus, the effect of column temperature was investigated. Excellent separation efficiency and the shortest analysis time were obtained when the column was thermostated at 35 °C, yielding narrow peaks with an average peak width at base (*w*_b_) of ~0.24 min. The effects of injection volume were investigated to improve the sensitivity without significant loss of resolution. Higher injection volumes yielded larger peak heights and areas, which translated to better sensitivity. However, peak widths also increased with the increase of the injection volume. Taking into account the peak shapes and band broadening, injection volume of 20 μL was selected.

**Figure 2:**
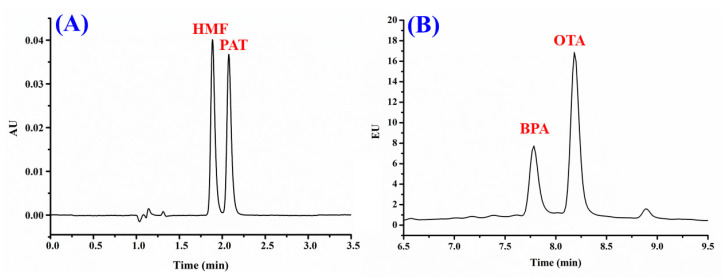
Typical chromatogram obtained from the introduction of standards mixture containing (**A**) 5-hydroxymethylfurfural (HMF; 100 ng mL^−1^) and patulin (PAT; 150 ng mL^−1^) using a photo-diode array detector; (**B**) bisphenol A (BPA; 20 ng mL^−1^) and ochratoxin (OTA; 45 ng mL^−1^) using a fluorimetric (FL) detector.

Although a fluorescence detector is many folds more sensitive than UV, the PAT and HMF exhibited weak fluorescence properties, and displayed strong UV absorption. Thus, absorbance wavelength at 276 nm and 284 nm was selected for PAT and HMF, respectively, for detection and quantification. OTA and BPA are fluorophores that displayed strong fluorescence properties; therefore, fluorescence detection was applied. Excitation and emission wavelengths were briefly optimised to obtain maximum responses for OTA and BPA. The chromatographic effluents were subjected to both photo-diode array (PDA) and fluorimetric (FL) detections that were connected in series to enable sensitive detection of all the analytes of interest. The adopted HPLC-DAD/FL conditions are summarised in Table 1, whilst Figure 2 shows typical chromatograms of the OTA, PAT, HMF, and BPA operated under the optimised conditions. The developed multi-contaminants analysis approach achieved separation of all solutes in less than 9 min, which is much shorter than some of the reported chromatographic approaches [12,19,30]. To date, this is the first report on the simultaneous separation and detection of OTA, PAT, HMF, and BPA in a single analysis.

### 3.2. Optimisation of Clean-Up and Preconcentration Strategies

The inherent complexity of fruit drinks and low levels of the analytes limits the direct analysis of the samples. Relatively high sugar (glucose, sucrose, xylose, and fructose) content in the fruit drinks also makes direct chromatographic analyses not possible [15]. Therefore, clean-up and preconcentration steps are required to overcome these problems. The first clean-up procedure involved the addition of Carrez solutions (a mixture of potassium hexacyanoferrate (III) and zinc acetate dihydrate) to the sample solutions followed by centrifugation to provide clarification effects. The precipitate was removed, and the pH of resulting sample solutions was adjusted to pH ~8 to remove excess Zn ions. The clarified solution was extracted separately using CHCl_3_ (to recover OTA, HMF, and BPA) and EA (to recover PAT) with vortexing, and the organic phases were collected and evaporated to dryness under a gentle stream of nitrogen. The dried residue was immediately reconstituted with 400 µL of MeOH:water (30:70; *v/v*) and filtered before injection. By using such clean-up approaches, interfering components are greatly reduced, which allows selective extraction of the targeted analytes.

### 3.3. Method Validation

#### 3.3.1. Linearity

A series of standard working solutions were prepared, extracted, and analysed under the optimised conditions outlined in Section 2.3 and Section 2.5. The maximum permitted level of each analytes was taken into consideration while selecting the concentration ranges for all the analytes. The regression equations and correlation coefficients are summarised in Table 2. Excellent linearities were achieved for all of the four analytes, with a correlation coefficient of *R* > 0.99.

#### 3.3.2. Limits of Detection and Quantitation

The LODs were determined with a signal to noise ratio of 3.3, whereas LOQs were calculated with a signal to noise ratio of 10. Relatively low LODs and LOQs were obtained for all analytes, as shown in Table 2. The LOD values obtained were 0.5 ng mL^−1^, 1.1 ng mL^−1^, 7.9 µg mL^−1^, and 1.0 ng mL^−1^ for OTA, PAT, HMF, and BPA, respectively. Overall, the achieved sensitivities meet the requirements for the assessment of these contaminants in the fruit drinks, with maximum permitted concentrations as defined by the European Union Commission and Codex Alimentarius. Interestingly, the developed HPLC-UV/FL method exhibited significantly lower LOD values for OTA (0.5 ng mL^−1^) and BPA (1.0 ng mL^−1^) compared to other reported methods using CE-PDA (OTA, 11.2 ng mL^−1^; BPA, 55 µg L^−1^) [31,32], and GC-MS (BPA, 20 µg L^−1^) [33], although the LOD values were comparable to those obtained using HPLC-MS/MS (OTA, 0.5 ng mL^−1^; BPA, 0.15 ng mL^−1^) [34,35].

#### 3.3.3. Precision and Accuracy Studies

The precision of the method was investigated by evaluating the repeatability and reproducibility of the method with three concentration levels (2, 20, and 40 ng mL^−1^ for OTA; 20, 60, and 150 ng mL^−1^ for PAT; 40, 400, and 800 ng mL^−1^ for HMF; 5, 40, and 80 ng mL^−1^ for BPA). Good intraday precision was obtained for both peak areas and retention time, with relative standard deviation values of <4% for both peak areas and retention, as indicated in Table S1. Accuracy studies of OTA, PAT, HMF, and BPA were performed in three replicates by spiking selected drink samples with a known amount of standard at different concentration levels (2, 20, and 45 ng mL^−1^ for OTA; 20, 60, and 150 ng mL^−1^ for PAT; 20, 400, and 800 ng mL^−1^ for HMF; 5, 40, and 80 ng mL^−1^ for BPA). Good recovery (Table S1) was achieved for all analytes, with values ranging from 82.73 ± 2.00% to 98.94 ± 1.81% indicating that the improved and optimised sample preparation and HPLC-PDA/FL methods are able to provide an acceptable quantitation of the contaminants in fruit drinks.

### 3.4. Application to Fruit Beverages and Drinks

**Figure 3:**
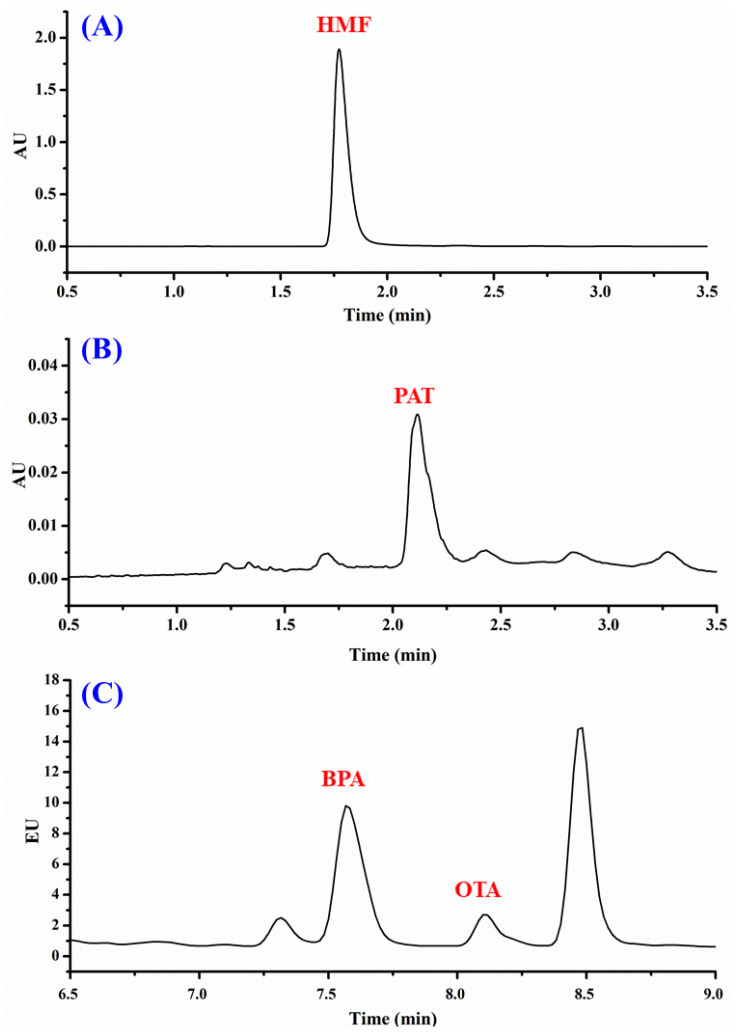
HPLC-PDA/FL analysis of fruit drink samples: (**A**) dates drink showing detected 5-hydroxymethylfurfural (HMF); (**B**) apple drink showing detected patulin (PAT); (**C**) ambarella drink showing detected bisphenol A (BPA) and ochratoxin A (OTA).

**Figure 4:**
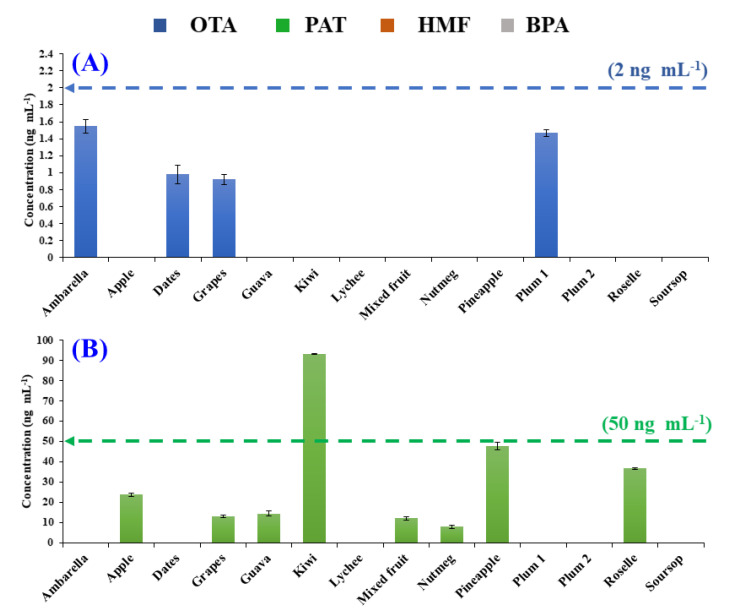

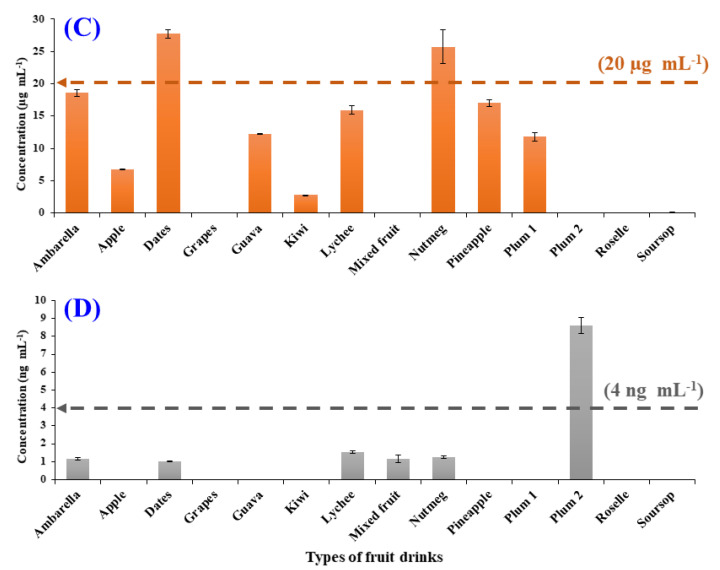
Graphical presentations of the positive fruit drink samples containing (**A**) ochratoxin A (OTA); (**B**) patulin (PAT); (**C**) 5-hydroxymethyfurfural (HMF); (**D**) bisphenol A (BPA). The coloured dashed lines correspond to the maximum permitted levels of the stated compounds in fruit juices established by the European Union (EU; for OTA, PAT, HMF) and the European Food Safety Authority (EFSA; for BPA).

The validated HPLC-PDA/FL method was applied to examine the occurrence and levels of OTA, PAT, HMF, and BPA in the selected commercialised fruit drink samples (Table 3). Figure 3 shows typical chromatograms obtained for some of the analysed samples (dates, apple, and ambarella). In this study, most of the analysed samples were derived from tropical fruits that are native to tropical and sub-tropical regions. Tropical fruits grow in a tropical climate which is humid and warm throughout the year, hence they are more susceptible to fungal infection [6]. In the current analysis, OTA (Figure 4A) was detectable in four samples: ambarella (1.55 ng mL^−1^), plum (1.47 ng mL^−1^), dates (0.98 ng mL^−1^), and grape drinks (0.92 ng mL^−1^). The occurrence of mycotoxins in the samples indicated that the fruits used for the preparation of the drinks had been infected with fungal growth. Interestingly, ambarella and plum indicated a higher level of OTA when compared with grape drinks. Such results signified that there was an increasing tendency of OTA contaminations in fruits from warmer and tropical climates, which are usually caused by *Aspergillus ochraceus*, as supported by other studies conducted by Afsah-Hejri et al. and Terra et al. [6,36]. The contamination of OTA in fruits from cool climates, on the other hand, is usually associated with *Penicillium verrucosum*. Although the highest concentration of OTA detected was 1.55 ng mL^−1^, it is still within the permitted level suggested by the European Union Commission (2 µg kg^−1^) [8].

For PAT, the current results (Table 3) indicated its occurrence at a different level in 57% of the analysed fruit drinks. In addition to the apple drink samples, the contamination of PAT by microfungi producing PAT was also observed at much higher levels in other fruit drinks. The highest PAT content was found in kiwi drink samples (93.28 ng mL^−1^), then pineapple drink samples (47.78 ng mL^−1^), followed by roselle (36.72 ng mL^−1^) and apple (23.8 ng mL^−1^) drink samples. The high concentration of PAT found in the kiwi drink is in good agreement with a similar study which reported 45.102–268.88 µg mL^−1^ of PAT in kiwi juice samples from different cultivars [37]. Surveys on the occurrence of PAT contamination in food mostly focus on apple-based products associated with the apple-rotting fungus *Penicillium expansum*; however, recently PAT was reported in various fruit commodities around the world [13,15]. Recent findings by Iqbal et al. (2018) indicated that 54.5% of the tested pineapple samples exceeded the permitted EU level (50 µg kg^−1^), with highest PAT content up to 460.3 µg kg^−1^ [13]. In order to mitigate the OTA and PAT contaminations, the selection of infected fruits must be strengthened, and cleaning must be improved prior to processing, as the applied thermal treatment cannot effectively remove the mycotoxins [11,12].

For HMF, 10 out of 14 samples tested positive, with concentrations ranging from 0.13–27.73 µg mL^−1^. The variation in the HMF contents may suggest that the fruit drink samples had undergone thermal treatment to improve sensory properties and extend their shelf life. In particular, dates (27.73 µg mL^−1^) and nutmeg (25.72 µg mL^−1^) samples surpassed the EU legal limit of 20 mg kg^−1^ (Figure 4C). Dates are a tropical fruit that are rich in nutrients and have high sugar content, mainly glucose and fructose. The presence of reducing sugar in an acidic environment could accelerate the generation of HMF via Maillard reaction [17,27]. Previous studies have reported the presence of HMF in dates syrup ranging from 42.8–84.2 mg/100 g dry weight [38], and approximately 1000 mg/kg were detected in dried dates [39].

BPA levels in the examined fruit drinks samples are given in Table 3, with average concentrations between 1.02–8.59 ng mL^−1^. Five of the positive samples showed lower BPA content than the maximum permitted EFSA limit (4 μg kg^−1^). The BPA chromatogram of the ambarella drink is illustrated in Figure 3C. The obtained values have good agreement in comparison with a previous study by Geens et al., which reported that the average BPA concentrations in beverages and canned foods were 1 ng mL^−1^ and 40.3 ng g^−1^, respectively [40]. In another study, the levels of BPA in juice samples were significantly higher, ranging from 40.27–82.55 μg kg^−1^ [30]. Nevertheless, the migration of BPA into food products varied depending on the food packaging type, as higher BPA migration was detected in canned food products in comparison to other types of food packaging [30,40,41,42]. Zhang et al. conducted a study on BPA prevalence in the environment by examining the presence of BPA in urine samples in several Asian countries. The average BPA detected in urine samples from Malaysia was 1.06 ng mL^−1^, whereby the daily BPA exposure of Malaysians was estimated to be 0.08–22.9 µg day^−1^ [25]. Thus, the obtained results are in accordance with previous investigations.

Although the levels of detected contaminants were mostly within the legal limits established by the accredited bodies (Figure 4), continuous monitoring of such contaminants in fruit drinks is important and necessary in order to safeguard consumers and protect public health. The current reported multi-contaminants analysis approach will be especially useful for routine quality control or monitoring of such contaminants in fruit juices and drinks. In the longer run, good agricultural and processing practices need to be strengthened to avoid fungal growth and mitigate the occurrence of these contaminants in fruit drinks.

## 4. Conclusions

This study described simple, rapid, and cost-effective preparation steps with HPLC-UV/FL detection to quantify OTA, PAT, HMF, and BPA in various fruit drinks. Using the optimised chromatographic conditions, the simultaneous separation of the four contaminants was achieved in <9 min. Validation data demonstrated good linearity, recoveries, retention, and response area reproducibility. The method was successfully applied for the determination of OTA, PAT, HMF, and BPA in various fruit drinks and concentrates. All the analysed samples indicated the presence of OTA (0.92–1.55 ng mL^−1^), PAT (7.93–93.28 ng mL^−1^), HMF (0.13–27.73 µg mL^−1^), and BPA (1.02–8.59 ng mL^−1^) at different concentration levels, either separately or in combinations. These observations signify the need for routine quality control of the commercialised fruit juices and drinks to confirm their quality and safety. The proposed multi-contaminants analysis approach provides considerable advantages in terms of cost, simplicity, analysis time, and sensitivity, and can be readily utilised in most food analytical laboratories for the routine monitoring and control of these contaminants.

## Figures and Tables

**Table 1:** Adopted HPLC operating conditions for simultaneous detection of 5-hydroxymethylfurfural (HMF), patulin (PAT), ochratoxin (OTA), and bisphenol A (BPA) using photo-diode array (PDA) and fluorimetric (FL) detectors.

Adopted HPLC Conditions		
Variable	Optimum Value	
Flow rate	1.2 mL min^−1^	
Temperature	35 °C	
Initial mobile phase composition (%)	MeOH: Acidified water (18:82)	
Gradient programming (Methanol composition, %)	0–2.0 min	(18%)
	2.0–8.0 min	(95%)
	8.0–10 min	(95%)
	10–10.5 min	(18%)
	10.5–12.5 min	(18%)
Injection volume	20 µL	
PDA configuration	HMF ^1^	284 nm
	PAT ^2^	276 nm
FL configuration	BPA ^3^	275 nm (excitation), 300 nm (emission)
	OTA ^4^	333 nm (excitation), 443 nm (emission)

^1^ HMF: 5-hydroxymethylfurfural; ^2^ PAT: patulin; ^3^ BPA: bisphenol A; ^4^ OTA: ochratoxin A.

**Table 2:** Calibration curves, limits of detection (LOD), and limits of quantification (LOQ) for ochratoxin A (OTA), patulin (PAT), 5-hydroxymethylfurfural (HMF), and bisphenol A (BPA) using the optimised HPLC-PDA/FL method.

Analytes	Linear Range (ng mL^−1^)	Regression Equation	Linearity, *r*^2^	LOD (ng mL^−1^)	LOQ (ng mL^−1^)
**OTA ^1^**	0.5–50	y = 4629.4x + 5522.6	0.9984	0.5	1.7
**PAT ^2^**	10–200	y = 77.499x − 57.109	0.9998	1.1	3.4
**HMF ^3^**	10–1000	y = 302.62x − 2243.6	0.9993	7.9	24.0
**BPA ^4^**	1–100	y = 8820.4x + 115511	0.9988	1.0	3.2

^1^ OTA: ochratoxin A. ^2^ PAT: patulin. ^3^ HMF: 5-hydroxymethylfurfural. ^4^ BPA: bisphenol A.

**Table 3:** Composition of ochratoxin A (OTA), patulin (PAT), 5-hydroxymethylfurfural (HMF), and bisphenol A (BPA) in the tested fruit drink samples using the optimised HPLC-PDA/FL method.

Types of Fruit Drinks	Average Concentration ± *SD* ^1^			
	OTA ^2^ (ng mL^−1^)	PAT ^3^ (ng mL^−1^)	HMF ^4^ (µg mL^−1^)	BPA ^5^ (ng mL^−1^)
Ambarella	1.55 ± 0.08	<LOD ^6^	18.59 ± 0.54	1.18 ± 0.07
Apple	<LOD	23.80 ± 0.82	6.80 ± 0.03	<LOD
Dates	0.98 ± 0.11	<LOD	27.73 ± 0.64	1.02 ± 0.01
Grapes	0.92 ± 0.06	13.06 ± 0.50	<LOD	<LOD
Guava	<LOD	14.53 ± 1.19	12.31 ± 0.06	<LOD
Kiwi	<LOD	93.28 ± 0.12	2.80 ± 0.01	<LOD
Lychee	<LOD	<LOD	15.94 ± 0.67	1.54 ± 0.06
Mixed fruit	<LOD	12.08 ± 0.94	<LOD	1.16 ± 0.19
Nutmeg	<LOD	7.93 ± 0.83	25.72 ± 2.60	1.25 ± 0.07
Pineapple	<LOD	47.78 ± 1.79	17.04 ± 0.49	<LOD
Plum 1	1.47 ± 0.04	<LOD	11.81 ± 0.66	<LOD
Plum 2	<LOD	<LOD	<LOD	8.59 ± 0.45
Roselle	<LOD	36.72 ± 0.46	<LOD	<LOD
Soursop	<LOD	<LOD	0.13 ± 0.01	<LOD

^1^*SD*: standard deviation. ^2^ OTA: ochratoxin A. ^3^ PAT: patulin. ^4^ HMF: 5-hydroxymethylfurfural. ^5^ BPA: bisphenol A. ^6^ < LOD: below limit of detection.

## Supplementary Materials

The following are available online at https://www.mdpi.com/2304-8158/9/11/1633/s1: Table S1: Intraday precision and accuracy of the developed HPLC-PDA/FL method.
