# Supplementary material for: Simultaneous Quantitative Assessment of Ochratoxin A, Patulin, 5-Hydroxymethylfurfural, and Bisphenol A in Fruit Drinks Using HPLC with Diode Array-Fluorimetric Detection
Source: Foods. 2020 Nov 9;9(11):1633. doi: 10.3390/foods9111633 (PMC7695309; doi:10.3390/foods9111633)
Supplement: Supplementary file 1 [file foods-09-01633-s001.pdf]

# Supplementary

**Table 1.** Intraday precision and **a**ccuracy of the developed HPLC-PDA/FL method.

| Analyte | Intraday precision | | | Accuracy | |
|---|---|---|---|---|---|
| | Concentration (ng mL-1) | RSD% (n=3) | | Concentration (ng mL-1) | Recovery % ± SD[2] (n=3) |
| | | RT[1] | Area | | |
| **OTA[3]** | **2** | 0.45 | 2.30 | 2 | 87.36 ± 1.81 |
| | 20 | 1.40 | 2.70 | 20 | 84.31 ± 2.72 |
| | 40 | 0.69 | 3.50 | 45 | 93.31 ± 2.22 |
| **PAT[4]** | 20 | 3.49 | 0.77 | 20 | 85.94 ± 2.22 |
| | 60 | 3.54 | 1.30 | 60 | 92.63 ± 1.90 |
| | 150 | 0.96 | 1.50 | 150 | 98.94 ± 1.81 |
| **HMF[5]** | 40 | 0.80 | 1.40 | 20 | 85.01 ± 2.00 |
| | 400 | 1.55 | 2.80 | 400 | 90.52 ± 1.63 |
| | 800 | 3.10 | 3.20 | 800 | 86.31 ± 2.22 |
| **BPA[6]** | 5 | 0.85 | 1.10 | 5 | 82.73 ± 2.00 |
| | 40 | 1.30 | 2.20 | 40 | 93.14 ± 1.63 |
| | 80 | 0.70 | 1.60 | 80 | 91.57 ± 2.22 |

[1] RT, retention time.

[2] SD, standard deviation.

[3] OTA, ochratoxin A.

[4] PAT, patulin.

[5] HMF, 5-hydroxymethylfurfural.

[6] BPA, bisphenol A.
